# Two-dimensional high resolution electron properties of femtosecond laser-induced plasma filament in atmospheric pressure argon

**DOI:** 10.1038/s41598-024-52868-w

**Published:** 2024-02-14

**Authors:** Junhwi Bak, Gerardo Urdaneta, Sagar Pokharel, Richard B. Miles, Albina Tropina

**Affiliations:** Department of Aerospace Engineering, Texas A&M University, College Station, TX 77845 USA

**Keywords:** Aerospace engineering, Applied physics, Plasma physics, Laser-produced plasmas

## Abstract

This work reports the measurement of two-dimensional electron properties over a nanosecond scale integration time across a femtosecond laser-induced plasma filament in atmospheric pressure argon. Radial electron properties across the $\sim 100$ $\upmu$m diameter filament are obtained at discrete axial locations at 2.5 mm steps by one-dimensional high-resolution laser Thomson scattering with a spatial resolution of 10 $\upmu$m. These measurements reveal plasma structural information in the filament. The Thomson spectral lineshapes exhibit clear spectral sidebands with an $\alpha$ parameter $\sim 1$, enabling the measurement of both electron temperature and density profiles. These measurements yield electron densities on the order of $10^{22}$/m^3^ and electron temperatures of $\sim 2$ eV. Heating from the probe laser due to inverse bremsstrahlung is taken into account to correct the Thomson scattering electron temperature measurements. Under these conditions, electron-neutral collision induced bremsstrahlung becomes the dominant laser-induced plasma heating process associated with the probe laser. The measurements reveal structural features of the filament, including an asymmetrically skewed density structure in the axial direction and reversed radial distributions of electron density and temperature.

## Introduction

Precision shape-controlled and well-localized nanosecond pulsed laser energy addition into a gas are both limited by the stochastic behavior of the laser-induced avalanche breakdown and by interactions of the evolving plasma with the laser radiation during the breakdown process. To overcome these limitations and provide versatile approaches for the creation of well-controlled energy addition, a dual femtosecond (fs) and nanosecond (ns) pulse approach is used. This approach takes advantage of a low-energy fs laser pre-pulse to create a pre-determined pattern which is subsequently energized and further shaped by the second high-power nanosecond laser pulse. Knowledge of the spatiotemporal properties of the initial femtosecond generated ionization and filament formation informs the optimum pulse shape, time delay, pulse duration and overlap of the second energy-depositing nanosecond pulse for specific applications such as aerodynamic flow control^1,2^, combustion control^3–5^, plasma tailoring^6–8^. Properties of interest include self-guiding, energy coupling, and plasma decay rates. This information provides guidance for optimizing the efficiency of the dual pulse energy deposition process and ensures a well-defined starting point for the high-power energy addition as well as the controlled selection of the desired final state. This approach enables precision timed and localized energy addition and can be extended to the formation of localized plasma and high temperature zones in air.

Over the past decade, there has been a growing interest in unraveling the properties of the fs laser-induced filaments. The experimental efforts have been geared towards the generation of filaments in different gases, such as atmospheric air, nitrogen, and argon^9,10^. In addition, different experimental techniques have been employed to obtain electron number density and temperature. Some of them include interferometry^9,11,12^, stark broadening^13^, in-line holography^14^, terahertz spectroscopy^11,15^, fluorescence^16^, and microwave diagnostics^10^. These works have mainly focused on the filament spatial^11–15^ and temporal^9–11,13^ profiles of electron number density. The dependence of electron number density as a function of laser pump energy^11,13,16^, pulse duration^13^, and focal length^16^ has been explored as well. The values obtained for the electron number density range between $10^{19}$ and $10^{24}$/m^3^. The reason for such a widespread range of densities lies in the different techniques, laser intensities, pulse energies, wavelengths, and gases used.

Understanding the spatial structure of filaments is important for the dual pulse applications mentioned above, as well as for other filament applications such as laser/microwave guiding^17,18^, and electric discharge guiding^19,20^. Additionally, spatially resolved results support the development and validation of multi-dimensional simulations^21–23^. It is worth noting that past experiments have often had limitations such as being indirect measurements of electron properties, having a low spatial resolution, or lacking electron temperature information. These limitations can be overcome with laser Thomson scattering (LTS). The Thomson scattering (TS) spectrum is a measure of the electron velocity distribution function, and it provides direct measurement of electron density and temperature with minimal assumptions on plasma conditions, such as symmetry, composition, and equilibrium. LTS has been widely implemented in high-temperature nuclear fusion plasmas, but has recently expanded its applications in various low-temperature plasma sources, including glow discharge^24^, arc discharge^25^, microwave discharge^26,27^, nanosecond pulsed discharge^28^, plasma jets^29^, and laser-induced plasmas^30–33^. When employing TS for low-temperature plasmas, it is essential to consider the effect that the probing laser has on the plasma itself. Such laser heating has been widely studied in various applications^30,31,34–36^.

One of the challenges of LTS application to the fs-generated plasma filament is the temporal decay of the plasma during the several nanosecond sampling pulse. This problem is minimized in argon filaments, which have a negligible temporal decay^9^ within the early few nanoseconds. However, we should note that the temporal decay will not ultimately limit the application of LTS to the filament. For molecular gases, even though there exists a sharp decay at the very early time $t<1$ ns, after that, the change over a few ns stays within the same order^9^. In this regime, a time window of a few ns can capture the decay dynamics in a temporally moving averaged manner^37^. Thus, LTS may not be ideal for the initial hundreds of ps scale, but will be useful to study temporal dynamics on the ns scale. The properties of plasma in the ns timescale hold significant importance for various applications mentioned earlier. Rather, a more practical issue with molecular gases is rotational Raman scattering (RRS), the spectrum of which overlaps the Thomson scattering for normal 90 degree collection geometries. To mitigate this issue, forward low angle Thomson scattering can be utilized. The fundamental principle is based on the frequency narrowing of the electron TS in the forward direction and the collection angle independence of RRS^38^.

In this work, electron properties across an argon filament that has $\sim 100$ $\upmu$m diameter are resolved with a resolution of 10 $\upmu$m, and structural characteristics are discussed. The use of argon eliminates the RRS but strong Rayleigh scattering is still present. The high-resolution one-dimensional (1D) LTS measurements are enabled by using a volume Bragg grating (VBG) filter^38^ which strongly suppresses the Rayleigh scattering and other interference at the probe laser wavelength. By repeating the measurement at 2.5 mm steps along the axial direction, this work reveals two-dimensional electron properties of the plasma filament. Direct measurement of electron Thomson spectra by LTS has also led to electron temperature distributions that have not been reported before. Following extraction of electron temperature information from the Thomson spectrum, inverse bremsstrahlung is taken into account in a time-dependent manner to correct for the plasma heating by the Thomson probing laser. Lastly, we note that the analysis of the results assumes a Maxwellian energy distribution of electrons^39,40^. The initial electron energy distribution function (EEDF) in the fs-generated filament channel is non-Maxwellian^41–43^ due to tunneling and multiphoton ionization processes. Electron-electron collisions are on the order of picoseconds, thus, the initial non-Maxwellian EEDFs complete their evolution to Maxwellian distributions in sub ns times.

## Results

### Filament image and filament length

**Figure 1:**
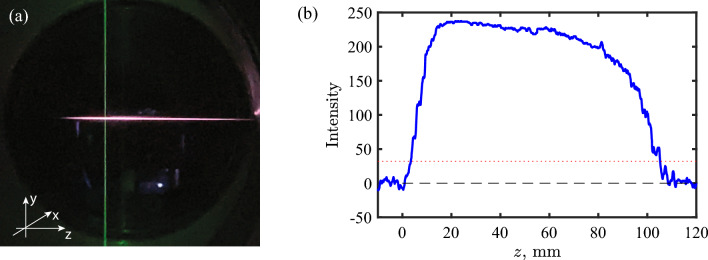
(**a**) An image of the femtosecond laser-induced plasma filament from atmospheric argon and a probing laser. (**b**) Intensity plot along the center line of the filament. The red dotted line indicates the $1/e^2$ intensity of the maximum intensity.

Figure 1 shows (a) an image of femtosecond laser-induced argon plasma filament at atmospheric pressure and the probing pulse perpendicular to the filament, and (b) a plot of the fluorescence intensity distribution along the filament center. From the intensity distribution, the length of filament $l_{\textrm{f}}$ ($1/e^2$ intensity) is approximately $l_{\textrm{f}}\,=\,101.5$ mm. The Rayleigh length $z_{\textrm{R}}$ is calculated to be $\sim 20$ mm using the fs laser wavelength $\lambda \,=\,829$ nm, the beam waist radius 80 $\upmu$m, and the $M^2$ factor 1.2. The observed filament length is approximately four times $z_{\textrm{R}}$ (or double the confocal distance), which indicates that a self-induced guiding structure is formed. Multiple filaments were not observed, based on the overall image intensity with a single well-defined maximum along the center line of the filament. This was confirmed by a single-shot filament imaging.

### Raw Thomson scattering spectra

Imaging of Thomson scattering across a filament is challenging due to its small diameter, typically $\sim$ 100 $\upmu$m. This size is an order of magnitude smaller than nanosecond pulse laser-induced breakdown plasmas, which have a size on the order of $\sim$ 1 mm. Thomson scattering has been widely used to understand the properties of those plasmas. For this work, the 1D LTS system using a VBG filter was implemented^39^ and the plasma distribution in the radial direction (in *y* direction) was resolved with a resolution of 10 $\upmu$m by binning two pixels along space. Axially, the filament was moved with a step length of 2.5 mm from 0 mm to 50 mm while the probing pulse remained fixed. The total travel length was 50 mm which is limited by the translation stage used.

**Figure 2:**
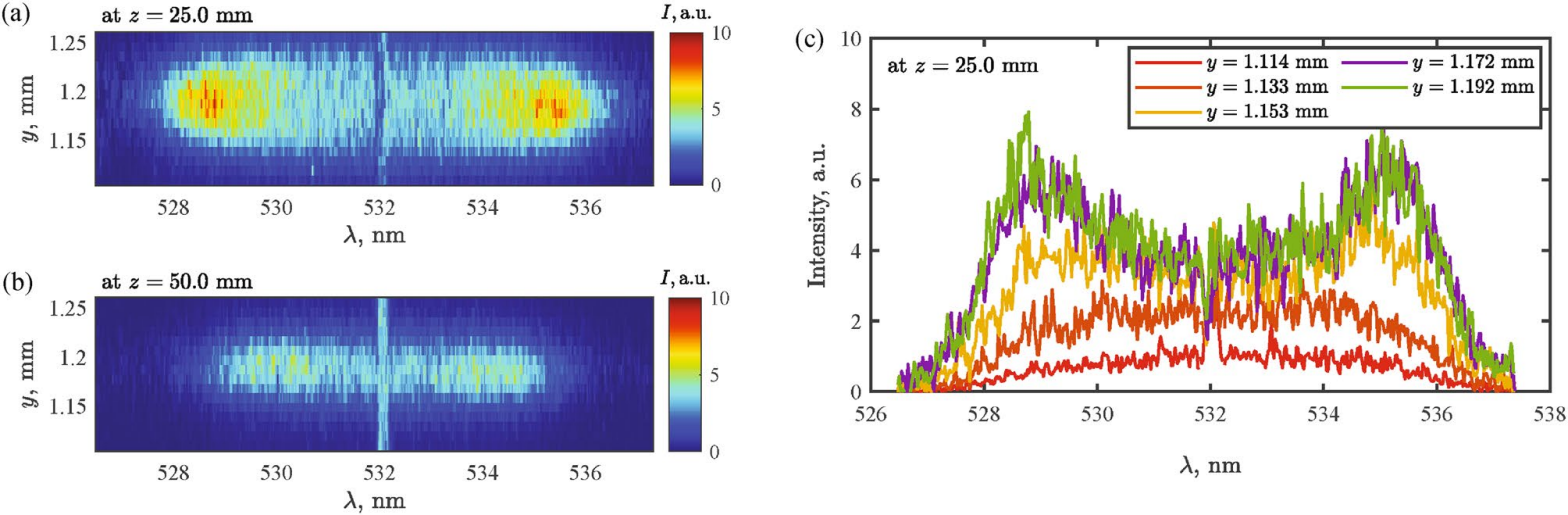
(**a**) Raw scattering spectra at $z=25$ mm. (**b**) Raw scattering spectra at $z=50$ mm. (**c**) Sample TS lineshapes at multiple *y* locations at $z=25$ mm.

Raw 1D scattering spectra and sample local TS spectra are shown as examples in Fig. 2 where (a) and (b) present raw scattering spectra at $z=25$ mm and at $z=50$ mm, respectively, and (c) shows sample local TS lineshapes at multiple *y* locations. It is worth noting that, in atmospheric pressure, the neutral density can be orders of magnitude greater than electron density, yielding significant Rayleigh scattering. It often causes a detector saturation, making the TS observation difficult. The results show that Rayleigh scattering and other stray light at the probe laser wavelength were successfully rejected. Note that none of the spectral portions were excluded in the raw spectra. Near the center of the filament, two sidebands become evident in the spectral lineshapes. The alpha parameter, defined as $\alpha \equiv 1/k \lambda _{\textrm{D}}$ where *k* is the incident light wavenumber and $\lambda _{\textrm{D}}$ is the Debye length, ranged from 0.7 to 1.4 at *z* = 25 mm and from 0.6 to 1.0 at *z* = 50 mm. A high alpha parameter is associated with scattering dominated by coherent ion-acoustic waves, which generate collective scattering side band features. The sidebands seen in the spectrum indicate that the density of electrons in the argon filament is high enough for the scattering to fall into the collective TS regime. This enables the spectrum to be fitted to extract both electron density and temperature. At each *y* location, a TS lineshape was fitted by collective Thomson fitting and the electron properties were extracted, which are presented in the next subsection. As pointed out earlier, the theory of electron TS lineshape used for the collective TS assumes Maxwellian electrons^39,40^. Considering that the electron-electron collision time of $\sim 1$ ps from the e-e collision frequency^44^ $\nu _{\textrm{ee}} \simeq 5\times 10^{-6} n_{\textrm{e}} \ln \Lambda / T_{\textrm{e}}^{3/2}$ /s as with $n_{\textrm{e}}\,=\,1\times 10^{23}$ /m^3^ and $T_{\textrm{e}}\,=\,2$ eV, temperature equilibrium will be reached in $\mathscr {O}(10)$ ps after several collisions. In a timescale of ns where TS is performed, the electrons are expected to follow the Maxwellian distribution.

### Electron properties distribution

After evaluating radial local electron properties by the collective fittings at each axial location, two-dimensional (2D) electron density $n_{\textrm{e}}$ and temperature $T_{\textrm{e}}$ maps were reconstructed. Note that the relative root mean square error (RRMSE) was used to quantify the signal-to-noise ratio (SNR) of the TS signals. The RRMSE is defined as $\text {RRMSE}\,=\,{\sqrt{\frac{1}{N} \sum \limits _{i=1}^{N} \left( X_i - f_i \right) ^2 }}/{ \bar{X}} \times 100\%,$ where $X_i$ and $f_i$ are the *i*th value of the experimental data and the fitted data, respectively. The overbar over a variable indicates the mean value. The data points having the RRMSE $>50\%$ were disregarded. The typical fitting error was $<10\%$.

**Figure 3:**
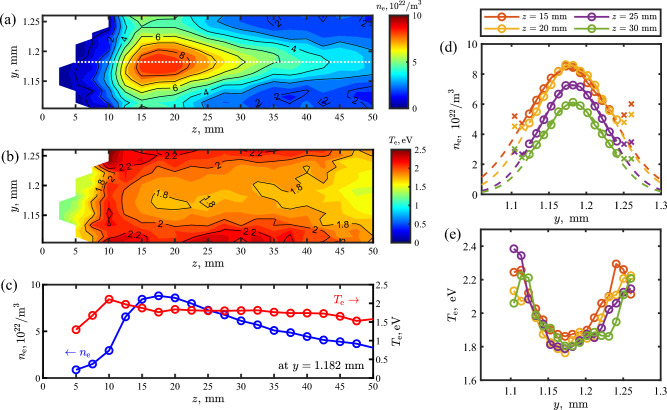
(**a**) Electron density $n_{\textrm{e}}$ map. Note that different spatial scales in *y* and *z* directions. (**b**) Electron temperature $T_{\textrm{e}}$ map. Electron heating is corrected. (**c**) Axial $n_{\textrm{e}}$ and $T_{\textrm{e}}$ distributions along the center of the filament at $y\,=\,1.182$ mm (along the white dotted line in (**a**)). (**d**) Radial $n_{\textrm{e}}$ distributions at $z\,=\,15$, 20, 25, and 30 mm. Fit curves in Gaussian are overlapped. When fitting, the points with the cross marks are excluded. (**e**) Radial $T_{\textrm{e}}$ distributions at $z\,=\,15$, 20, 25, and 30 mm.

Figure 3 shows extracted plasma structure information of the atmospheric argon filament. Figure 3a,b present the 2D distributions of electron density and temperature. Electron density on the order of $10^{22}$/m^3^ is in good agreement with other reported work with comparable fs pulse parameters^9,45^. For the electron temperature, laser heating from the probe pulse by electron-ion inverse bremsstrahlung and electron-neutral inverse bremsstrahlung was evaluated and corrected. Details on the heating correction can be found in the Methods section. Axial $n_{\textrm{e}}$ and $T_{\textrm{e}}$ distributions along the center of the filament are shown in Fig. 3c. A sharp gradient of $n_{\textrm{e}}$ at the head of the filament and an elongated low gradient towards the tail of the filament are observable. Along the filament, $T_{\textrm{e}}$ decreases relatively slowly compared to $n_{\textrm{e}}$. In Fig. 3d,e, radial $n_{\textrm{e}}$ and $T_{\textrm{e}}$ distributions at multiple *z* locations from 15 to 30 mm in 5 mm steps are plotted, respectively. In Fig. 3d, fitted curves in the Gaussian form, thus, $n_{\textrm{e}}(y)=a\exp {\left( -(y-b)^2/c^2 \right) }$, where *a*, *b*, and *c* are the fitting parameters, are overlapped. Note that the data points with the cross marks are excluded in the fitting process as the values at these points are erratically overestimated due to the low signal-to-noise. This exclusion is further justified by confirming that the total area underneath the spectral lineshape, which is proportional to $n_{\textrm{e}}$, is monotonically decreasing toward the radial edge of the filament. The density distributions closely follow a Gaussian distribution. It should be noted that the size of the probing laser beam is comparable to that of the filament, and the observed plasma information is the integration in depth (*x* direction) over the probing beam waist. Since the spatial distributions in the dimensions across the filament are assumed to be Gaussian, an inverse Abel transform does not need to be performed to extract the spatial information. Lastly, when comparing Fig. 3d,e, one can observe that an electron temperature dip is formed where the density is high. Such reversed distribution between the temperature and density is similar to the distribution found in a microwave plasma jet^29^.

## Discussion

The length of the filament is approximately four times the Rayleigh length, suggesting a self-induced guided filament was formed. The elongated filament that is longer than the Rayleigh length suggests there is the plasma de-focusing effect that extends the filament length. Additionally, we note that, in this work, the fs laser is focused down using a convex lens, thus, it is pre-focused filamentation. The pre-focused filamentation has different characteristics compared to the freely propagating fs filamentation; the pre-focused filament has a relatively short propagation length with a filament diameter on the order of 100 $\upmu$m generated from a laser intensity on the order of $10^{17}$–$10^{18}$ W/m^2^ while the freely propagating filament has a diameter on the order of 1 mm and the intensity on the order of $10^{16}$ W/m^2^^46^. The observed filament in this work has a diameter of 160 $\upmu$m and an intensity $I_\mathrm{max} = 1.4 \times 10^{18}$ W/m^2^, which falls well within the common pre-focused filament characteristics range^4^. The Gaussian beam peak power $P\,=\,2\times$ $2.9$ mJ/100 fs = 58 GW is above the critical power for self-focusing and filamentation of the Gaussian beam, $P_\mathrm{cr}=3.77 \lambda ^2 / (8 \pi n_2 n_0)=7.36$ GW for argon using the formula from Ref.^47^. However, there were no apparent multiple filaments (across the cross-section of the fs pulse), indicating a single self-focusing filament was formed. This may be attributed to the pre-focused filamentation where the focused beam diameter may be too small to develop well-separated multiple filaments within its own dimension, unlike the case of the freely propagating fs pulse.

Propagation and filamentation of the femtosecond laser pulse are dictated by an equilibrium between the plasma defocusing effect, which is determined by the multiphoton/tunnel ionization rates, and the focusing Kerr effect caused by the non-linearity of the refractive index dependence on the laser intensity. Analytically, the critical intensity in the femtosecond laser filament can be found from the balance of the Kerr nonlinearity of the medium and a defocusing effect that is produced by the induced plasma. This balance is given as follows^48^,

1 $$\begin{aligned} n_2 I_\mathrm{L}\cong \frac{\tau _\mathrm{p}}{2 n_{\textrm{e}}^\mathrm{cr}} R(I_\mathrm{L}), \end{aligned}$$

where $n_2=1.1\times 10^{-23}\,\hbox {m}^{2}$/W is the Kerr coefficient for argon^49,50^, $R(I_\mathrm{L})$ is the production rate of electrons, $\tau _\mathrm{p}\,=\,100$ fs is the pulse duration, and $n_{\textrm{e}}^\mathrm{cr}\,=\,1.04\times 10^{24}$ /m^3^ is the critical plasma density. Based on the calculated values of the ionization probability in argon using the PPT (Perelomov–Popov–Terent’ev) theory^51–53^, the expression for $R(I_\mathrm{L})$ can be written in a form following a power law, $R(I_\mathrm{L})= n_\mathrm{Ar} R_0 (I_\mathrm{L}/I_0 )^\beta$, where $n_\mathrm{Ar}=2.45 \times 10^{25 }$ /m^3^ is the argon neutral density at atmospheric pressure conditions and $R_0=1.66 \times 10^{4}$ /s is the ionization probability at the laser intensity $I_0=1.2 \times 10^{17}$ W/m^2^. The exponent of the power law $\beta \,=\,8$ was chosen to approximate the calculated probability dependence on the intensity in the range of laser intensities from $I_0$ to $I_\mathrm{max}=1.4 \times 10^{18}$ W/m^2^. The $I_\mathrm{max}$ was estimated using experimental parameters of the laser pulse. A solution of the Eq. (1) gives the critical laser intensity $I_\mathrm{L}^\mathrm{cr}=2.1 \times 10^{17}$ W/m^2^. Neglecting the defocusing effect, avalanche ionization, and recombination losses due to the short duration of the pulse, the maximum number density of electrons generated by the fs pulse can be evaluated using the following equation,

2 $$\begin{aligned} \frac{dn_{\textrm{e}}}{dt} = R_\mathrm{i}(n_\mathrm{ar}-n_{\textrm{e}}), \end{aligned}$$

where $R_\mathrm{i}$ is the ionization rate at the specific laser intensity. At the laser intensity $I_\mathrm{max}$, $R_\mathrm{i}=9.4\times 10^{12}$ /s, and after 0.5 fs the electron number density reaches the critical value $n_{\textrm{e}}^\mathrm{cr}=1.04 \times 10^{24}$ /m^3^, where defocusing effect starts to play a role. Thus, we can evaluate the number density of electrons generated by 100 fs pulse in argon assuming that the laser intensity is equal to $I_\mathrm{max}$ at time $t\le 0.5$ fs and the laser intensity is equal to the critical laser intensity $I_\mathrm{L}^\mathrm{cr}$ after that. As a result, the estimated electron number density generated by the 100 fs pulse in argon in the focus is $n_{\textrm{e}}^\mathrm{calc}=1.1\times 10^{23}$ /m^3^, which closely matches the measured peak density of $n_{\textrm{e}}=0.9\times 10^{23}$ /m^3^.

**Figure 4:**
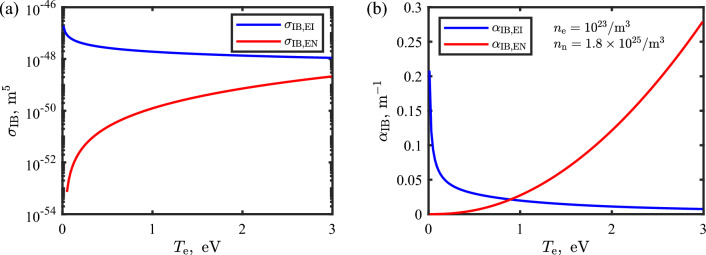
(**a**) Electron-ion and electron-neutral inverse bremsstrahlung cross-sections, $\sigma _\mathrm{IB,EI}$ (blue) and $\sigma _\mathrm{IB,EN}$ (red), as a function of electron temperature. (**b**) Electron-ion and electron-neutral inverse bremsstrahlung absorption coefficients, $\alpha _\mathrm{IB,EI}$ (blue) and $\alpha _\mathrm{IB,EN}$ (red), as a function of electron temperature. For the calculation of the coefficient, $n_{\textrm{e}}\,=\,10^{23}$/m^3^ and $n_\mathrm{n}\,=\,1.8\times 10^{25}$/m^3^ were used.

For LTS it is important to correct the measured electron temperature results to account for plasma heating by the probing laser. This is usually dominated by inverse bremsstrahlung heating. Note that the electron density is less affected than the electron temperature unless the induced heating is significant^36^ or the introduced laser intensity exceeds the breakdown threshold. For this work, we assume that the measured electron density needs no correction. In applications of LTS to thermal plasmas, plasma heating by electron-ion inverse bremsstrahlung is dominant^30,36,54,55^. LTS is widely used in laser-induced plasmas as well^31–34^, and there exist multiple works on heating consideration by inverse bremsstrahlung^31,35,36^. For LTS applications to thermal/laser-induced plasma sources, only electron-ion inverse bremsstrahlung was considered^31,32^. It is commonly acceptable to consider only electron-ion inverse bremsstrahlung, because electron-neutral inverse bremsstrahlung cross-section $\sigma _\mathrm{IB,EN}$ is two orders of magnitude smaller than electron-ion inverse bremsstrahlung cross-section $\sigma _\mathrm{IB,EI}$, see Fig. 4a. (Details on the cross-section calculations are given in the Methods section.) Additionally, in thermal equilibrium plasmas, the ionization degree when the electron temperature is even a few electron volts approaches 1, so the ratio between neutral and electron density $n_\mathrm{n}/n_{\textrm{e}}\gtrsim \mathscr {O}(1)$, so electron-ion inverse bremsstrahlung is dominant. However, in the fs-generated filament at atmospheric pressure, the ionization fraction is low and the bulk neutral density barely changes, so $n_\mathrm{n}/n_{\textrm{e}}\gtrsim \mathscr {O}(10^2)$ is often satisfied. This leads to both electron-ion and electron-neutral inverse bremsstrahlung becoming comparable, or electron-neutral inverse bremsstrahlung even being a dominant process. This is shown in Fig. 4b where electron-ion and electron-neutral inverse bremsstrahlung absorption coefficients, $\alpha _\mathrm{IB,EI}$ and $\alpha _\mathrm{IB,EN}$, are plotted as a function of electron temperature. In this calculation, $n_{\textrm{e}}\,=\,10^{23}$/m^3^ and $n_\mathrm{n}\,=\,1.8 \times 10^{25}$/m^3^ were used, corresponding to an ionization fraction of $0.5\%$. Additionally, it is noteworthy that $\alpha _\mathrm{IB,EI}$ and $\alpha _\mathrm{IB,EN}$ have an opposite dependency on $T_{\textrm{e}}$. The electron-neutral inverse bremsstrahlung absorption has a positive curvature on $T_{\textrm{e}}$, which can accelerate the heating process. Thus, it is important to take into account the electron-neutral inverse bremsstrahlung in plasma heating in LTS measurements of atmospheric plasma filaments, corresponding to a weakly ionized plasma.

The Gaussian distribution of the density arises from the Gaussian intensity distribution of the focused fs laser. The main mechanisms responsible for plasma formation are multiphoton-ionization (MPI) and tunnel ionization. The probability of ionization is understood to be proportional to some exponential power of the local laser intensity, which relies on the composition of neutrals and the laser wavelength^52^. Considering the femtosecond timescale of plasma generation, the losses in electron density during the laser pulse can be neglected, leading to an electron density profile following a power law with respect to the intensity. Note that the power of a Gaussian distribution remains Gaussian. The good agreement of the density profile to a Gaussian fit function indicates the preservation of the underlying physics in the density structure.

The reversal tendency of electron temperature along the radial direction seen in Fig. 3e is similar to that which was reported in the argon atmospheric microwave plasma jet^56,57^. It was suggested that the increase in $T_{\textrm{e}}$ may be due to the balance of the large recombination with a higher ionization rate that requires an increase in $T_{\textrm{e}}$ toward the edge^56^. The similar electron density and temperature structures in different plasma sources may indicate that underlying physics between different plasma sources may share common characteristics, which could be interesting follow-up research. Another hypothesis can be as follows. The electron temperature is a measure of the average energy of electrons. It changes primarily due to the flux of electrons into the control volume (transport processes) and various sources/sinks of energy^58^. When radiation and transport are neglected, assuming relatively uniform plasma at the core and during small time scales, the electron temperature is governed by the electron energy conservation equation, Eq. (3),

3 $$\begin{aligned} \frac{\partial }{\partial t}\left( \frac{3}{2} n_{\textrm{e}} k_{\textrm{B}} T_{\textrm{e}}\right) = - {Q}_\mathrm{ET} - {Q}_\mathrm{EV} + {Q}_\mathrm{VE} + {Q}_\mathrm{CR}. \end{aligned}$$

Elastic collisions, represented by $Q_\mathrm{ET}$, encompass both Coulomb collisions ($\nu _\mathrm{ei} \propto n_{\textrm{e}}$) and electron-neutral collisions ($\nu _\mathrm{en} \propto n_\mathrm{n}$). As a result, $Q_\mathrm{ET}$ exhibits a dependence on both $n_{\textrm{e}}^2$ and $n_{\textrm{e}}$^6,59^. In contrast, inelastic collisions involve various processes such as chemical reactions $Q_\mathrm{CR}$, where three-body recombination having an electron as the third body leads to $n_{\textrm{e}}^2$ dependence, and other electron impact reactions have $n_{\textrm{e}}$ dependence. Additionally, excitation, $Q_\mathrm{EV} \propto n_{\textrm{e}}$, contributes to the energy exchange when vibrationally excited energy levels are accessible. Superelastic collisions $Q_\mathrm{VE}$ make a minor contribution, only become dominant when $T_\mathrm{v} > T_{\textrm{e}}$. The chemical reaction term plays a crucial role when electronically excited species strongly engage in energy exchange with electrons. However, its contribution in the fs-induced plasma is relatively weak compared to other processes. Hence, the balance between $Q_\mathrm{ET}$ and $Q_\mathrm{EV}$ is crucial in determining the electron temperature profile. The dominance of the excitation term $Q_\mathrm{EV}$ occurs when vibrationally excited energy levels are accessible. Species like $\textrm{N}_2$, with numerous vibrational levels, make significant contributions to the electron energy balance, while $\textrm{Ar}$ lacks vibrational excitation. In argon plasma as in the present work, the Coulomb collisions prevail, resulting in a sharp relaxation, thus the dip of electron energy at the center, due to its $n_{\textrm{e}}^2$ dependence. Conversely, in other plasmas such as $\textrm{N}_2$, $Q_\mathrm{EV}$ assumes importance and may result in sustained high electron energy at the core.

## Methods

### Experimental setup

**Figure 5:**
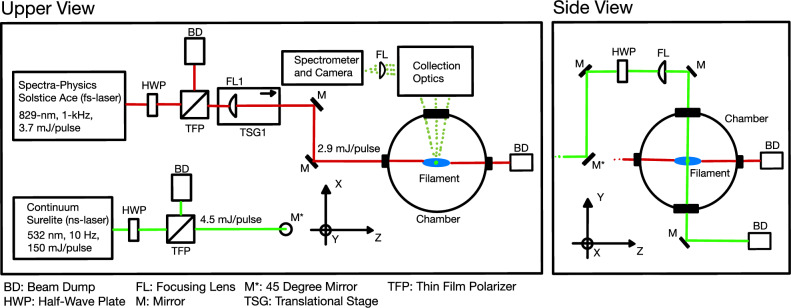
A schematic of the experimental setup.

A schematic of the experimental setup is presented in Fig. 5. A femtosecond laser (Spectra-Physics Solstice Ace) was used to produce the plasma filament. The laser pulse was set at 829 nm and was operated with the repetition rate 1 kHz with a pulse duration of $\sim 100$ fs and 2.9 mJ per pulse. The fs laser has a beam diameter of 10 mm. The fs pulse was focused down by a plano-convex lens having a 1, 000 mm focal length. The lens was set on a translation stage that has a total 50 mm travel length. In repetitive filamentation, the cumulative effect, such as filament spectrum, supercontinuum generation, gas heating and localized depletion of neutral density, could occur depending on various conditions; laser wavelength, pulse energy, focusing condition, pulse duration, etc.^60–63^. For the fs pulse parameters in the present work, the gas recovery is complete within 1 ms^62^, thus, no cumulative effect occurs between the fs pulses. An Nd:YAG 532 nm nanosecond laser (Continuum Surelite EX) was used as a probe laser beam. The probing laser was operated without an injection seeding at the repetition rate of 10 Hz, with a pulse energy of 4.5 mJ and a pulse full-width half max (FWHM) duration of 7 ns. The probing laser was focused by a plano-convex lens having a 500 mm focal length. The relative temporal delay between two laser pulses was set by a delay generator (Stanford Research Systems DG-535); the fs pulse peak being 3 ns ahead of the ns pulse peak. Jitters in the experiment were characterized by capturing scattered lights of the fs and ns pulses from a viewport. The typical jitter between the fs and ns pulses was $\pm 1.1$ ns. Note that the important jitter factor is that of the fs pulse with respect to the observation time gate window because the plasma condition within the gate window is governed by the fs pulse. A Q-switch sync-output from the ns laser triggers a camera, thus, the gate window. The jitter between the sync-output and the fs pulse was $\pm 0.5$  ns. By passing the probing pulse across the filament, the 1D distribution of electron properties along *y* direction (along the radial direction of the filament, see Fig. 5) was obtained at each axial *z* direction. The filament was moved axially by moving the fs pulse focusing lens on a translation stage, the total 50 mm in 2.5 mm steps.

For high-resolution 1D measurement, Thomson scattering using a reflecting volume Bragg grating (VBG) notch filter was performed. The VBG notch filter had a typical spectral rejection bandwidth 5 cm^-1^ ($\approx 150$ GHz) and was designed for 532 nm (OptiGrate BNF-532). The Bragg filter provides transmittance of the unfiltered wavelengths up to 80%, while achieving optical density (OD) of 4 for the unwanted light. Note that both Thomson and Rayleigh scattering are centered at the laser wavelength (without a drift velocity) as the spectral broadening originates from the Doppler shifts. The Rayleigh scattering has a linewidth of an order of 1–10 GHz, which falls within the filter’s rejection bandwidth. Angle tuning of the VBG filter optimizes the rejection of Rayleigh and stray probe laser light allowing high-resolution 1D measurement. Details of the principle and method can be found in Ref.^38^. The probing laser is introduced from the top of the chamber (the *y* direction, see Fig. 5.), and the scattering is collected through one of the side viewports in *x* direction, thus, the collection angle is $90^{\circ }$. The object plane where the filament lies was relayed by a $f\,=\,300$ mm and a $f\,=\,200$ mm 2” diameter achromatic lenses. The rays were collimated by a 1” diameter *f* = -50 mm achromatic lens into the VBG filter for spectral filtering of near-532 nm light. The image was rotated by a set of two mirrors forming a periscope and was focused by a 1” $f\,=\,200$ mm plano-convex lens into the vertical slit of a spectrometer (Princeton Instruments IsoPlane 320). The total image magnification of the system was 2.67. The scattering signal was captured by an emiCCD camera (Princeton Instruments PI-MAX4:1024EMB) with a gate opening of 5 ns and a gain of $\times 1,000$. A total of 2, 000 laser shots were accumulated for one raw spectrum image. The spatial scattering data were binned by two pixels, giving the final spatial resolution of 10 $\upmu$m in *y* direction.

### Plasma heating correction

The use of a laser pulse for plasma diagnostics can induce plasma heating due to the interaction between the photons and electrons. The laser heating in the Thomson scattering experiment has been widely investigated over decades^55,64^, in various plasmas such as laser-induced breakdown plasmas^31,34,65^, and thermal plasmas^36^. Neglecting cooling effects, the influence of laser heating of the plasma can be quantified by the change of electron temperature^31,36,54,66,67^ as follows,

4 $$\begin{aligned} \frac{\Delta T_{\textrm{e}}}{T_{\textrm{e}}} = \frac{2}{3} \frac{\alpha _\mathrm{IB} F_\mathrm{L}}{k_{\textrm{B}} T_{\textrm{e}} n_{\textrm{e}}}, \end{aligned}$$

where $\alpha _\mathrm{IB}$ is the inverse bremsstrahlung absorption coefficient which includes electron-ion inverse bremsstrahlung $\alpha _\mathrm{IB,EI}$ and electron-neutral inverse bremsstrahlung $\alpha _\mathrm{IB,EN}$. $k_{\textrm{B}}$ is the Boltzmann constant, $F_\mathrm{L}$ is the laser fluence, $T_{\textrm{e}}$ is the electron temperature, and $n_{\textrm{e}}$ is the electron density.

Assuming only the singly ionized ions, the electron-ion inverse bremsstrahlung absorption coefficient $\alpha _\mathrm{IB,EI}$ is expressed as^68^,

5 $$\begin{aligned} \alpha _\mathrm{IB,EI} = \sigma _\mathrm{EI} n_{\textrm{e}} n_\mathrm{i} G(\lambda ,T_{\textrm{e}}) (1 - e^{- h c/ (k_{\textrm{B}} T_{\textrm{e}} \lambda )}), \end{aligned}$$

where $\sigma _\mathrm{EI} = \frac{4}{3} \left( \frac{2 \pi }{ 3 m_{\textrm{e}} k_{\textrm{B}} T_{\textrm{e}}} \right) ^{1/2}\left( \frac{ e^2 }{ 4 \pi \varepsilon _0} \right) ^{3} \frac{ \lambda ^3}{m_{\textrm{e}} c^4 h}$ is the electron-ion absorption cross-section, the factor $(1 - e^{- h c/ (k_{\textrm{B}} T_{\textrm{e}} \lambda )})$ is a correction term for stimulated emission^68^, and $G(\lambda ,T_{\textrm{e}})$ is the Gaunt factor. $\sigma _\mathrm{EI}$ is obtained from averaging over the electron velocity space assumed to follow a Maxwellian distribution^68^. $G(\lambda ,T_{\textrm{e}})$ for $\lambda =532$ nm laser is obtained as $G(T_{\textrm{e}})=1.086+4.86\times 10^{-6}T_{\textrm{e}}-1.213\times 10^{-11}T_{\textrm{e}}^2$ by reading the figures in Ref.^69^. For the conditions of the present work, the Gaunt factor is around 1.1–1.2.

The electron-neutral inverse bremsstrahlung absorption coefficient $\alpha _\mathrm{IB,EN}$ is given in a similar form by,

6 $$\begin{aligned} \alpha _\mathrm{IB,EN} = \sigma _\mathrm{EN} n_{\textrm{e}} n_\mathrm{n} (1 - e^{- h c/ k_{\textrm{B}} T_{\textrm{e}} \lambda }), \end{aligned}$$

where the absorption cross-section $\sigma _\mathrm{EN}$ is given as $\sigma _\mathrm{EN}(T_{\textrm{e}}) = 9.60\times 10^{-5} T_{\textrm{e}}^2 A(T_{\textrm{e}}) \lambda ^3$, where $T_{\textrm{e}}A(T_{\textrm{e}})$ is a smooth function in $T_{\textrm{e}}$^68^, and is fit for argon from Table I in Ref.^70^; $T_{\textrm{e}}A(T_{\textrm{e}})=3.066\times 10^{-33}+2.776\times 10^{-36}T_{\textrm{e}}+2.727\times 10^{-40}T_{\textrm{e}}^2$.

It is worth noting that Eq. (4) provides the upper limit of laser heating because it takes into account the full probing pulse energy. Recall that the plasma filament is created 3 ns before the ns pulse temporal peak and that the ns pulse FWHM duration is $\tau _\mathrm{FWHM}\,=\,7$ ns. Therefore, the laser heating only starts from the moment the filament is created at $t_\mathrm{0}$. We evaluate the time-dependent heating for the temporal laser fluence profile $F_\mathrm{L}(t)$, which is approximated as a Gaussian profile in this work, thus, $F_\mathrm{L}(t) = F_0 \frac{1}{\sigma \sqrt{2 \pi }}\exp (-\frac{t^2}{2\sigma ^2})$ where $F_0=E_\mathrm{L}/(\pi r_\mathrm{L}^2)$ with a probing laser pulse energy $E_\mathrm{L}\,=\,4.5$ mJ and a laser beam radius $r_\mathrm{L}\,=\,70$ $\upmu$m, and $\sigma \,=\,\tau _\mathrm{FWHM}/ 2 \sqrt{2\ln 2}$ with a pulse duration $\tau _\mathrm{FWHM}\,=\,7$ ns. The maximum fluence over the temporal profile is $3.9\times 10^{4}$ J/m^2^. Using the probing pulse parameters, the maximum intensity of the probe beam is obtained as $I_\mathrm{max}^\mathrm{probe}=8 \times 10^{13}$ W/m^2^. Then, the electron temperature increase from the initial temperature $T_\mathrm{e0}$ is given as,

7 $$\begin{aligned} T_{\textrm{e}}(t) = T_\mathrm{e0} + \frac{2}{3 k_{\textrm{B}} n_{\textrm{e}}} \int _{t_\mathrm{0}}^{t} \alpha _\mathrm{IB}(t)F_\mathrm{L}(t)dt. \end{aligned}$$

Defining the camera gate opening time $t_\mathrm{i}$ and closing time $t_{\textrm{f}}$, the perceived electron temperature during the TS detection is then for a time interval $t=[t_\mathrm{i},t_{\textrm{f}}]$. We assume that the measured temperature $T_{\textrm{e},\textrm{exp}}$ is the temporal mean temperature while the camera gate is open, thus, Eq. (7) is solved in an iterative manner starting with an initial guessed $T_\mathrm {e_\mathrm{0}}$ until the condition $\frac{1}{t_{\textrm{f}} - t_\mathrm{i}}\int _{t_\mathrm{i}}^{t_{\textrm{f}}} T_{\textrm{e}}(t)dt=T_{\textrm{e},\textrm{exp}}$ is satisfied. Note that other cooling or heating mechanisms such as collisional cooling or recombination heating are not included as their influences on electron temperature and density over a few ns in the atmospheric argon filament are negligible^9^, thus, the laser heating is assumed to be the dominant mechanism. Recalling the electron density is less influenced during the heating process^30^, the experimentally obtained $n_{\textrm{e},\textrm{exp}}$ was used as a constant. The process was repeated at each location, and the found initial electron temperature $T_\mathrm {e_\mathrm{0}}$ was provided in Fig. 3b. Over the resolved region, the heating correction $T_\mathrm {e_\mathrm{0}}/T_{\textrm{e},\textrm{exp}}$ was 65–75%.

**Figure 6:**
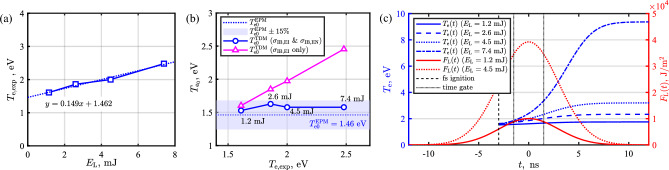
(**a**) $T_{\textrm{e},\textrm{exp}}$ as a function of a probe energy. The unperturbed electron temperature $T_\mathrm{e0}$ is obtained by the extrapolation. (**b**) Temperature correction comparison between the time-dependent method and the extrapolation method. For the time-dependent algorithms, two plots are made; one where both $\alpha _\mathrm{IB,EI}$ and $\alpha _\mathrm{IB,EN}$ are taken into account (solid blue circle), and one only with $\alpha _\mathrm{IB,EI}$ (solid magenta triangle). (**c**) Calculated $T_{\textrm{e}}(t)$ curves (blue) at each probing laser energy and two sample $F_\mathrm{L}(t)$ profiles (red).

We also note that the reliability of the above-described time-dependent heating correction was compared to the typical extrapolation correction method^71,72^ where the probing energy is scanned to obtain the unperturbed electron temperature at the zero energy by the extrapolation. Figure 6a shows $T_{\textrm{e},\textrm{exp}}$ at pulse energy of $E_\mathrm{L}\,=\,1.2$, 2.6, 4.5, and 7.4 mJ. By the extrapolation method (EPM), the unheated temperature $T_\mathrm {e_\mathrm{0}}^\mathrm{EPM}$ = 1.46 eV was obtained. The time-dependent method (TDM) was applied to each measured electron temperature at each probing energy, and the corrected temperature at each probing energy by the time-dependent method, $T_\mathrm {e_\mathrm{0}}^\mathrm{TDM}$, was compared to the temperature by the extrapolation, $T_\mathrm {e_\mathrm{0}}^\mathrm{EPM}$, in Fig. 6b. In Fig. 6b, for the time-dependent algorithms, two corrections were made; one where both $\alpha _\mathrm{IB,EI}$ and $\alpha _\mathrm{IB,EN}$ are taken into account (solid blue circle), and one only with $\alpha _\mathrm{IB,EI}$ (solid magenta triangle). When both electron-ion and electron-neutral inverse bremsstrahlung absorption are taken into account, all corrected values were within $15\%$ of that obtained from the extrapolation method. This indicates that the time-dependent method can provide consistent correction results. The exclusion of the electron-neutral inverse bremsstrahlung cannot properly address the laser heating, indicating the necessity of electron-neutral inverse bremsstrahlung absorption. In Fig. 6c, calculated $T_{\textrm{e}}(t)$ curves (blue) at each probing laser energy and two sample $F_\mathrm{L}(t)$ profiles (red) are given. The fs pulse comes at $t\,=\,-3$ ns and the time gate opening is 3 ns. Note that the time gate of 3 or 5 ns did not make a noticeable difference, so, in the main experiment, the 5 ns gate was used for more signal collection. Lastly, it is needless to say that too high probing pulse energy can induce non-negligible electron density increase due to strong heating^36^, which should be avoided. Increase in the measured density from the one ($3.72\times 10^{22}$ /m^3^) at the minimum used energy of 1.2 mJ was $1\%$ ($3.76\times 10^{22}$ /m^3^) at 2.6 mJ, $9\%$ ($4.05\times 10^{22}$ /m^3^) at 4.5 mJ, and becomes $>40\%$ ($5.29\times 10^{22}$ /m^3^) at 7.4 mJ. Such a drastic density increase at 7.4 mJ is attributed to the accelerated heating due to the enhanced electron-neutral inverse bremsstrahlung absorption along with temperature increase as can be seen in Fig. 6c as well. Note that the heating dependency on $n_{\textrm{e}}$ is in $\alpha _\mathrm{IB,EI}$, but not in $\alpha _\mathrm{IB,EN}$. So, when $\alpha _\mathrm{IB,EN}$ is dominant, the choice of the probing pulse energy is a trade-off between the density perturbation and the signal-to-noise. In this work, 4.5 mJ was used for probing pulse energy.

## Data Availability

The datasets generated during and/or analysed during the current study are available from the corresponding author on reasonable request.
